# The anti-inflammatory effects of oridonin in resisting esophageal cancer

**DOI:** 10.3389/fonc.2025.1585057

**Published:** 2025-06-18

**Authors:** Mengfan Peng, Xiaofang Zhang, Lei Yang, Baosong Liu

**Affiliations:** ^1^ Faculty of Medicine, Huanghuai University, Zhumadian, Henan, China; ^2^ Department of Pharmacy, Zhumadian Traditional Chinese Medicine Hospital, Zhumadian, Henan, China; ^3^ Key Laboratory of Cardiovascular and Cerebrovascular Diseases, Tianfang Pharmaceutical Co., Ltd., Zhumadian, Henan, China

**Keywords:** esophageal cancer, oridonin, TLR4/NF-κB/NLRP3, inflammatory factor, peripheral blood cells

## Abstract

**Objectives:**

Explore whether Oridonin (Ori) improves esophageal cancer by interfering in the TLR4/NF-κB/NLRP3 inflammasome.

**Materials and methods:**

An esophageal mouse model was induced by 4-nitroquinoline N-oxide (4-NQO) for 16 weeks. Starting from the 17th week of modeling, the mice were randomly divided into three groups: the model group (M), the high dose group of Ori (Ori -H) and the low dose group of Ori (Ori -L). The weight, diet, and water intake of the mice were recorded at the end of the experiment. H&E staining was used for esophageal tissue to evaluate pathological status. The tumor markers, inflammatory factor, mRNA and protein expression of TLR4/NF-κB/NLRP3 inflammasome related indicators in serum and esophageal tissue was determined by ELISA, qPCR, and western blot (WB) respectively. The blood cell analyzer was used for measuring the proportion of various blood cells.

**Results:**

Ori can increase the weight, the intake amount of food and water of mice (*P*<0.05, *P*<0.01). In parallel, Ori can alleviate pathological changes of esophageal tissue, decrease the levels of inflammatory factor tumor necrosis factor α (TNF-α), interleukin-1β (IL-1β), cyclooxygenase-2 (COX-2), and interleukin-6 (IL-6) in serum (*P*<0.01), and down-regulate granulocyte (Gran), Gran-to- Lymphocyte (Lymph) ratio (NLR), monocyte (Mon)-to-lymph ratio (MLR), and platelets-to-Lymph ratio (PLR) in the peripheral blood, while increasing Lymph, red blood cell (RBC), hemoglobin (HGB) (*P*<0.05, *P*<0.01). Moreover, the protein expression of toll-like receptor 4 (TLR4), phosphorylated nuclear factor-κB (p-NF-κB), IL-1β, NOD-like receptor hot protein domain related protein 3 (NLRP3), aspartate specific cysteine protease-1 (Caspase-1), apoptosis-associated speck-like protein (ASC), N-cadherin, and p-GSK3β was significantly inhibited by Ori (*P*<0.05, *P*<0.01), and the mRNA expression of proliferating cell nuclear antigen (PCNA), Ki67, and B-cell lymphoma-2 (Bcl-2) was significantly inhibited, while Bax mRNA was increased by Ori (*P*<0.05, *P*<0.01).

**Conclusion:**

This study provides evidence indicating that Ori may inhibit inflammatory response by inhibiting TLR4/NF-κB/NLRP3 inflammasome activation, ultimately exert anti esophageal cancer effects.

## Introduction

Esophageal cancer (EC) is a malignant tumor of the digestive tract formed by dysplasia of the esophageal epithelium. Due to atypical early symptoms, most patients are diagnosed in the middle to late stage, and EC usually forms micrometastasis in the early stage. Therefore, only 10% of EC patients can receive surgical treatment at the time of treatment (1). According to China’s cancer burden data in 2022 released by the National Cancer Center (NCC), EC is one of the five major causes of death of cancer patients in China, and both the incidence rate and mortality are higher than the global average (2–4). Notably, the pathological process of esophagitis and EC is consistent with the hypothesis that the “tumor originates from chronic inflammation” which was raised by Virchow, a German pathologist in 1863. Tumor related inflammation is considered the “seventh marker of cancer” (5), with approximately 1/5 of cancer patients experiencing long-term chronic inflammatory stimulation, and 4/5 of cancer patients also experiencing inflammatory cell infiltration into cancer tissue (6). During the occurrence and development of EC, various risk factors such as overheated diet, gastroesophageal reflux, obesity, smoking, alcohol consumption, etc. can cause mucosal damage and chronic inflammation, leading to continuous damage and regeneration of normal squamous epithelial cells in the esophageal mucosa. Persistent inflammation causes an increase in the release of various pro-inflammatory neurotransmitters, promotes cell growth and invasion, induces mutations, increases angiogenesis, and ultimately develops into tumors, further leading to a vicious cycle (7, 8). Therefore, exploring the intervention effect of drugs on EC from the perspective of inflammation is of great significance.

Inflammasomes, as key step driving the inflammatory response, play an important role in the progression of tumors. Among them, NLRP3 inflammasome is the most representative and widely studied “star molecule”, which is a protein complex composed of NLRP3, Pro-caspase-1, and ASC. Numerous research has suggested that the activation of NLRP3 inflammasomes plays the role of oncogenes in EC (9, 10). Existing studies have confirmed that TLR4 is a mediator of EC cell proliferation and a target for EC treatment (11). Transcription factor NF-κB is downstream of the TLR4 and is the main regulator of inflammation, mediating cytokine storms. The increase in TLR4 expression is associated with the activation of NF-κB, in response to TLR4 ligands, promoting an increase in pro-inflammatory cytokine levels, and mediates the occurrence of inflammatory diseases (12). Meanwhile, as NF-κB transcription increases, NLRP3 inflammasomes are activated as multiprotein complexes in the cytoplasm (13). After assembly, pro-inflammatory caspase-1 precursor proteins are activated into caspase-1, thereby promoting the secretion of inflammatory factors (14), establishing a chronic inflammatory environment to provide conditions for the proliferation, invasion, and migration of EC cells, ultimately promoting the occurrence and development of EC.

There is no name for EC disease in traditional Chinese medicine (TCM). According to its clinical manifestations, it can be classified as “choking diaphragm”. TCM scholars throughout history have believed that the etiology of “choking diaphragm” is complex, often due to qi stagnation, blood stasis, phlegm coagulation, and other obstruction of the esophagus. Over time, heat and toxin have developed, infiltrating and corroding the esophagus (15). Therefore, Chinese medicine intervention in EC often uses products that clear heat, detoxify, and promote blood circulation and collaterals. The plant of *Rabdosia rubescens (Hemsl.)* Hara belongs to the liver, lung, and stomach meridians, which is used in Chinese medicine for its effect of clearing heat and detoxicating, activating blood circulation, and relieving pain (16). Oridonin (Ori) is a representative anti-cancer component of *Rabdosia rubescens (Hemsl.)* Hara, which has various biological activities such as anti-inflammatory, anti-tumor, anti-oxidant, and anti-hypertensive. The anti-tumor and anti-inflammatory effects of Ori have been widely studied in various animal models, such as colon cancer (17), lung cancer (18), as well as acute lung injury (19), vascular inflammation (20), and other animal models. There are studies reporting that Ori is a specific inhibitor of NLRP3 inflammasomes (21). Existing research indicates that the heat toxicity in TCM is closely related to inflammation in modern medicine. However, the pharmacological or mechanism research of Ori anti-tumor focused on proliferation, apoptosis, autophagy, migration and invasion, and less from the perspective of inflammation. And, there is currently no experimental study on whether Ori can intervene in the activation of TLR4/NF-κB/NLRP3 inflammasomes to treat EC. Therefore, we make the TLR4/NF-κB/NLRP3 inflammasome as the main line, supplemented by serum inflammatory factors, peripheral blood cell typing, tumor cell proliferation and apoptosis indicators to explore the anti-EC effect of Ori in this study.

## Materials and methods

### Materials

4-NQO, Sigma-Aldrich (batch no. N8141-5G, Shanghai, China). Ori, Hengcheng Zhiyuan Biotechnology Co., Ltd (batch no. N1700, Chengdu, Sichuan, China). Mouse IL-1β, mouse IL-6, mouse TNF-α, and mouse COX-2 ELISA test kits, Calvin (batch no. CK-E20533M, CK-E20012M, CK-E20220M, and CK-E93152M, Suzhou, Jiangsu, China). Anti-NLRP3, anti-NF-κB, anti-ASC, and anti-p-NF-κB, Bioss biotechnology Co., Ltd (batch no. bs-6655R, bs-0465R, bs-6741R, and bs-3543R, Beijing, China). Anti-E-cadherin, anti-Caspase-1, anti-N-cadherin, anti-β-actin, anti-TLR4, anti-GSK3β, and anti-p-GSK3β, Proteintech (batch no. 22018-1-AP, 22915-1-AP, 20874-1-AP, 20536-1-AP, 19811-1-AP, 22104-1-AP, and 67558-1-lg, Wuhan, Hubei, China). Anti-IL-1β, Servebio (batch no. GB11113, Wuhan, Hubei, China). FastPure Cell/Tissue Total RNA Isolation Kit V2, Vazyme-innovation in enzyme technology (batch no. RC112, Nanjing, Jiangsu, China). HyperScript RT SuperMix for qPCR and HyperScript 2 × SYBR Green qPCR Master Mix were purchased from APExBIO (batch no. K1074 and K1070, Houston, USA). Bicinchoninic acid (BCA) protein concentration detection kit, and 5 × Protein loading buffer were purchased from Solarbio (batch no. PC0020, and P1040, Beijing, China).

### Animals and treatments

The SPF-grade C57BL/6 male mice (6 weeks old) were purchased from the Jinan Pengyue Experimental Animal Breeding Co., Ltd., Shandong, China (license number: SCXL(Lu)20190003, laboratory animals certificate number: 370726221100428743);. Animals were housed under standard conditions in a breeding room (20-25°C, relative humidity, 50-60%, 12-h dark/light cycle) with standard chow and water ad libitum. Axperimental animal ethics Batch number: DWLL202206044, all procedures involving mice comply with the ethics of life sciences at Henan University of Chinese Medicine.

Preparation of cancer inducing agent 4-NQO solution: Dissolve 4-NQO in propylene glycol to prepare a storage solution of 5 mg/mL and store it at 4°C. When using it, take 1mL of the storage solution and add 49 mL of sterilized distilled water to prepare a 100 μg/mL 4-NQO aqueous solution. Place it in a drinking bottle for mice and let the mice drink it freely. Change it once a week.

After a week of adaptive feeding, 40 mice were randomly divided into control (Con, 10 mice) and EC groups by the random number table method. The Con group of mice drank normal drinking water, while the mice in the EC groups were given drinking water containing 4-NQO (100 μg/mL) for 16 weeks. Then the mice were given normal drinking water. Starting from the 17th week of modeling, the model preparation mice were randomly divided into the model group (M), high dose group of Ori (Ori -H, 80 mg/kg) and low dose group of Ori (Ori -L, 40 mg/kg) (17, 22), with 10 mice in each group. Drug intervention began in the 17th week of the experiment and lasted for 7 weeks (23, 24). The Con group of mice given the same volume of solvent. After the experiment is completed, samples are taken sequentially for indicator testing. The general steps are to take blood for peripheral blood cell counting. After blood collection, separate the serum and perform biochemical index testing. Finally, esophageal tissue was collected for pathological examination and mRNA and protein detection of relevant indicators.

### Body weight, diet and water consumption

On the last day of the experiment, record the weight, water, and diet intake of the mice.

### Hematoxylin and eosin staining

After taking out the esophageal, take photos and record the appearance changes. Then the esophagus tissue was fixed in 4% cell tissue fixative. After fixation, embed in paraffin. Esophagus tissue sections (5μm thick) were stained with H&E. After scanning with a digital slice scanner (Ningbo Jiangfeng Bioinformatics Technology Co., Ltd, KF-PRO-005EX, Zhejiang), observe the pathological changes in esophageal tissue.

### Biochemical parameter analysis

After the experiment, all mice were fasted for 12 hours before blood collection. Whole blood was dropped into the reagent tube containing EDTAK2 anticoagulant, and measure the proportion of RBC, HGB, Mon, Lymph, and Gran in the whole blood in the “pre-dilution” mode by the blood cell analytical apparatus. Then, the NLR, MLR, and PLR were calculated. Part of the whole blood was collected in a 1.5mL centrifuge tube, left at 4 °C for 15-30 minutes, centrifuged, and the supernatant (serum) was collected, IL-1β, TNF-α, IL-6, and COX-2 levels were determined using ELISA kits.

### Quantitative Real-time PCR

Selecting *β-actin* as an internal reference, PCR technology was used to detect the mRNA levels of IL-1*β*, TNF -*α*, IL-6, COX-2, Bax, Bcl-2, PCNA, and Ki67 in esophageal tissue. The primers used were designed and synthesized by Wuhan Sevier Biotechnology Co., Ltd. and Zhengzhou Dianjing Technology Co., Ltd. Calculated the relative expression by using the 2^-ΔΔCt^ method. The following oligonucleotides were used as PCR primers: *IL-1β* forward: GCATCCAGCTTCAAATCTCGC, *IL-1β* reverse: 5′-TGTTCATCTCGGAGCCTGTAGTG-3′; *TNF-α* forward: 5′-CCCTCACACTCACAAACCACC-3′, *TNF-α* reverse: 5′-CTTTGAGATCCATG CCGTTG-3′; *IL-6* forward: 5′-CCCCAATTTCCAATGCTCTCC-3′, *IL-6* reverse: 5′-CGCACTAGGTTTGCCGAGTA-3′; COX-2 forward: 5′-GAAATATCAGGTCA TTGGTGGAGA-3′, COX-2 reverse: 5′-ATGCTCCTGCTTGAGTATGTCG -3′; *BAX* forward: 5′-CCCGAGA GGTCTTTTTCCGAG-3′, *BAX* reverse: 5′- CCAGCCCAT GATGGTTCTGAT-3′; *Bcl-2* forward: 5′-GGTGGGGTCATGTGTGTGG-3′, *Bcl-2* reverse: 5′-CGGTTCAGGTACTCAGTCATCC-3′; *PCNA* forward: 5′-GTCGGGTG AATTTGCACGTA-3′, *PCNA* reverse: 5′-CTCTATGGTTACCGC CTCCTC-3′; *Ki67* forward: 5′-AATCTGTGGAAGAGCAGGTTAGC-3′, *Ki67* reverse: 5′-TCCTGGGA GGCAGTCTTCAT-AG-3′; *β-actin* forward: 5′-CACCCA GCACAATGAAGATC AAGAT-3′, *β-actin* reverse: 5′-CCAGTTTTTAAATCCTG AGTCAAGC-3′.

### Western blot

Total protein in esophageal tissue was extracted using RIPA lysis buffer which containing protease inhibitor and phosphoprotease inhibitor. After thorough lysis, the supernatant was collected by centrifugation at 12000 rpm/min and 4°C for 10 min. Subsequently, the BCA assay kit (Epizyme, Shanghai, China) was used to measure the protein concentration in the supernatant, and each sample was adjusted to a uniform concentration. The corresponding volume of 5 × loading buffer was added in a 1:4 ratio according to the instructions and boiled at 95°C for 6 min. 10 μg protein was electrophoresed on polyacrylamide gels and electroblotted onto PVDF membranes. After the membrane transfer is completed, place the PVDF membrane in 5% skim milk powder and seal it for 2 hours. Then incubated with anti-NLRP3 (1:1500), anti-GSK3β (1:800), anti-IL-1β (1:1000), anti-TLR4 (1:1000), anti-Caspase-1 (1:1500), anti-ASC (1:1000), anti-NF-κB (1:1000), anti-E-cadherin (1:1000), anti-p-NF-κB (1:1000), anti-N-cadherin (1:2000), and anti-p-GSK3β (1:1000) at 4°C for 4-12h. All antibodies were diluted with primary antibody diluent. The PVDF membranes were incubated with secondary antibody (Bioss, Beijing, China) at dark conditions for 1 h, imaged with two-color infrared laser imaging system (LICOR, Brasilia, USA), and performed grayscale measurement via ImageJ v. 1.51.

### Statistical analysis

Data were analyzed with SPSS v. 21.0 for Windows and expressed as the mean ± standard deviation (SD). Fisher’s least significant different method in one-way analysis of variance was used to test the homogeneity of variance, the Games-Howell method was used when the variances were uneven, and the Bonferroni post-test was used for comparison between two groups. And *P* < 0.05 was considered statistically significant.

## Results

### Ori improved body weight loss and increased the consumption of diet and drinking water in 4-NQO induced EC mice

**Figure 1:**
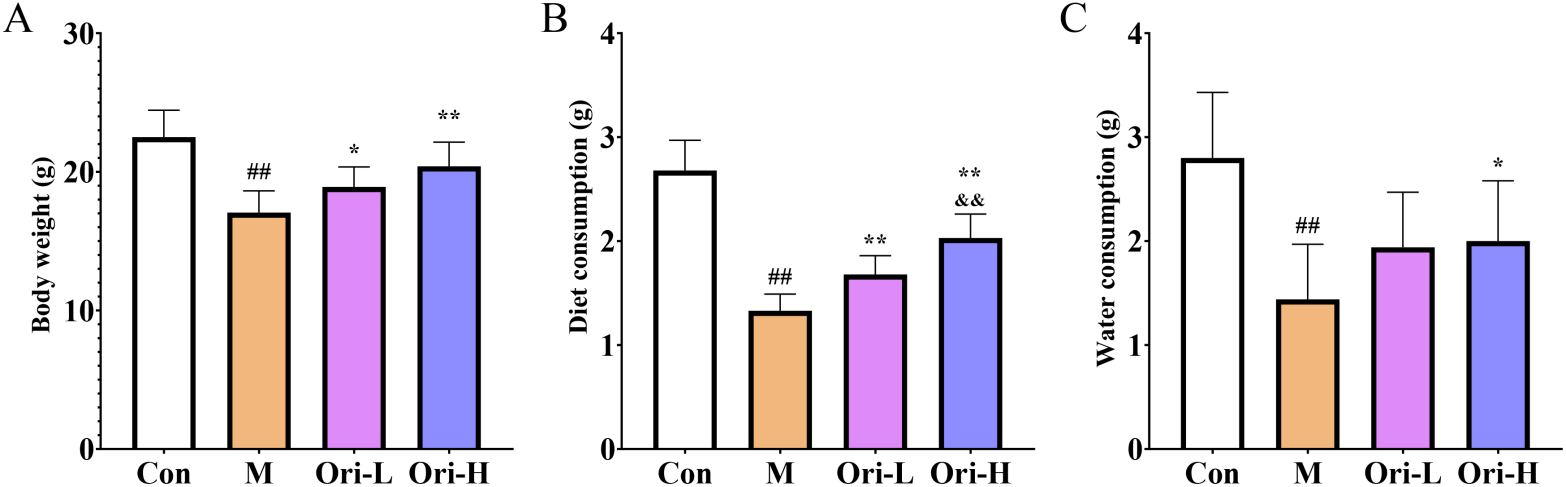
Ori improved body weight loss and increased the consumption of diet and drinking water in mice **(A)** Ori improved body weight loss in mice. **(B)** Diet measurement of mice. **(C)** Measurement of drinking water in mice. Data were expressed as means ± SD (*n* = 9-10), the Games-Howell method was used for comparison between two groups. ^##^ *P* < 0.01 vs the Con; *P < 0.05 vs the M; **P < 0.01 vs the M; ^&&^ *P* < 0.01 Ori-H vs the Ori-L.

During the whole experiment, one mice in the M group died at 18 W, one mice in the Ori-L group died at 22 W, indicating that intervention with Ori can prolong the survival time and reduce their mortality rate of EC mice. By the end of the experiment, the Con group mice had shiny hair, normal activity, and rapid response; The M group mice gradually became thin in shape, slow in movement, disheveled and matte in hair, accompanied by phenomena such as arched back, curling up, and blurred eyes; The status of mice in each drug intervention group was between the Con group and the M group. However, some mice had disheveled and matte hair, and their movements were relatively slow. At the end of the experiment, we tested the weight, diet, and water intake of mice, so as to observe the impact on the quality of life of mice more intuitively. As shown in **Figure 1**, the weight, diet, and water intake of mice in the M group were significantly reduced than that in the Con group (*P* < 0.01). Relative to the M group, the weight and the diet consumption of mice increased significantly in the Ori-L group (*P*<0.05 or *P* < 0.01), the weight and the diet consumption of mice increased significantly in the Ori-H group (*P* < 0.01). However, relative to the M group, the Ori-L group showed no significant improvement in drinking water, while the Ori-H group significantly increased the amount of drinking water in mice (*P* < 0.01). Relative to the Ori-L group, the diet consumption of mice increased significantly in the Ori-H group (*P* < 0.01). The above results indicate that Ori can improve the diet and water intake of EC mice to a certain extent, increase their weight, and thus improve their quality of life.

### Ori alleviated the histopathological changes of esophagus tissue in 4-NQO induced EC mice

**Figure 2:**
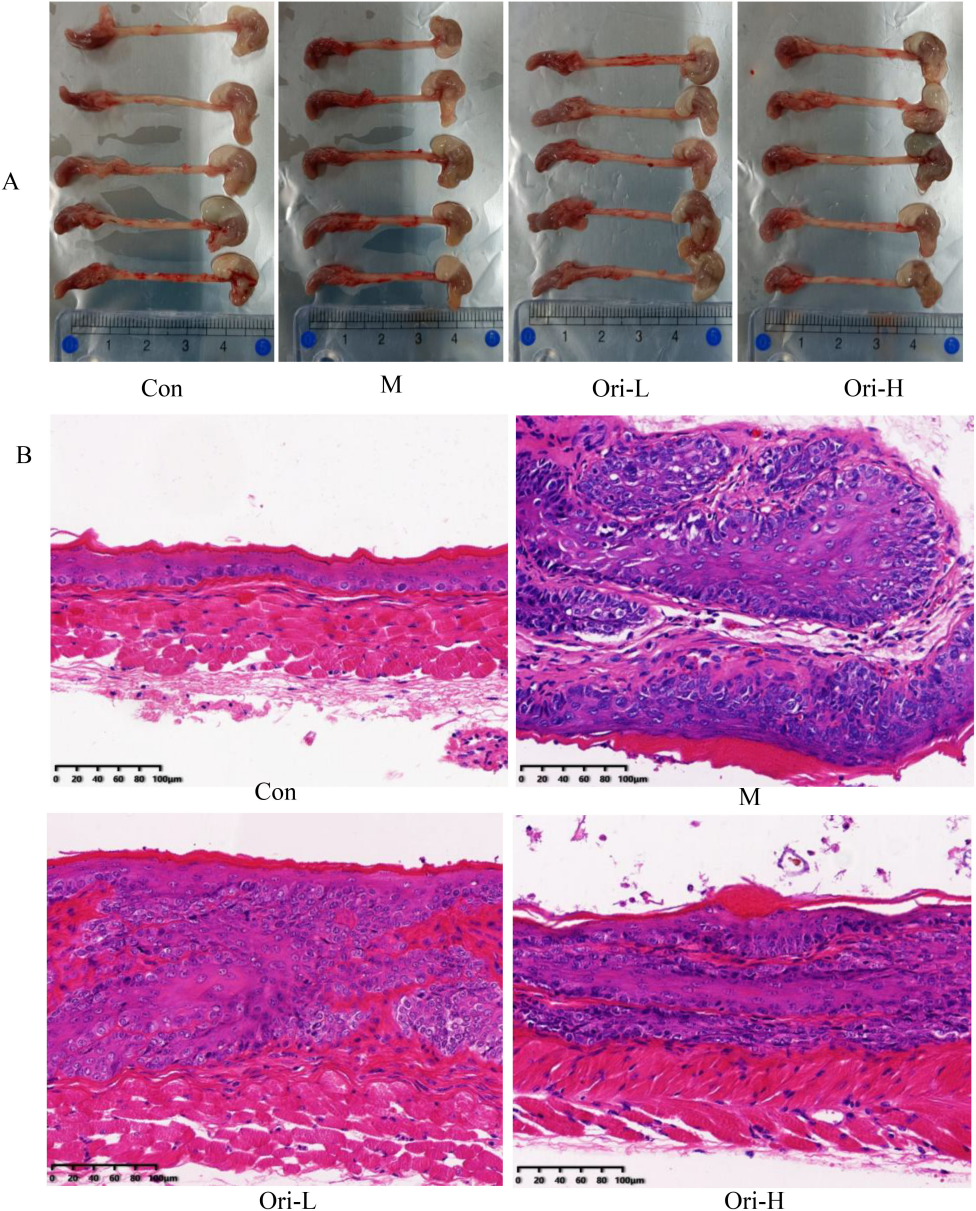
Ori alleviated the histopathological changes of esophagus tissue in mice. **(A)** Photos of representative gross esophagi with tongue and stomach. **(B)** Esophageal tissue (× 200) in H&E staining. Bars = 100 μm.

This work found that the apparent changes of the esophageal tissue in mice with EC were significant. As shown in **Figure 2A**: In the Con group, the length of esophageal tissue was around 3.5 cm, and the esophagus was smooth and slender without swelling. The esophageal tissue of mice in the group M was significantly shorter than that in the group Con, with a length of about 2.5 cm, and the esophagus became shorter and thicker. The length of esophageal tissue in the Ori-L and Ori-H groups was about 3.0 cm, and there was no bulge or swelling under the naked eye.

Then HE staining results in **Figure 2B** indicate that Ori can reduce the degree of esophageal tissue cancer in mice: For the Con group, the esophageal mucosa of mice was normal, including about 1-3 layers of basal/upper basal cells, 3-4 layers of the intermediate layer, surface squamous cells and keratinized layer. The cells were arranged neatly and no apparent abnormal cells or pathological mitosis was identified under the field of vision. For the M group, esophageal squamous cell carcinoma was formed in the esophageal mucosa of mice, which was mainly characterized by the irregular and close arrangement of polygonal, round and fusiform tumor cells. Pathological mitosis was common, and there was infiltration of a large number of inflammatory cells. For the Ori-L group, the normal structure of the esophageal mucosa was disrupted, with scattered distribution of irregular cells. Cells in the basal layer of the esophagus showed moderate to severe hyperplasia. Some of them presented cancerous sites, while others showed the formation of cancer nests. For the Ori-H group, the normal epithelial structure of esophageal tissue was disrupted, arranged in disorder, and the levels were significantly increased. The main manifestations of cell morphology are varying in size and shape, but cell atypia is relatively small, nuclear atypia, increased nuclear to cytoplasmic ratio, rare pathological mitotic phase, and mostly manifested as basal cell proliferation. In some areas, low-level intraepithelial neoplasia can be seen.

### Ori improved peripheral blood cell count of EC mice induced by 4-NQO

**Figure 3:**
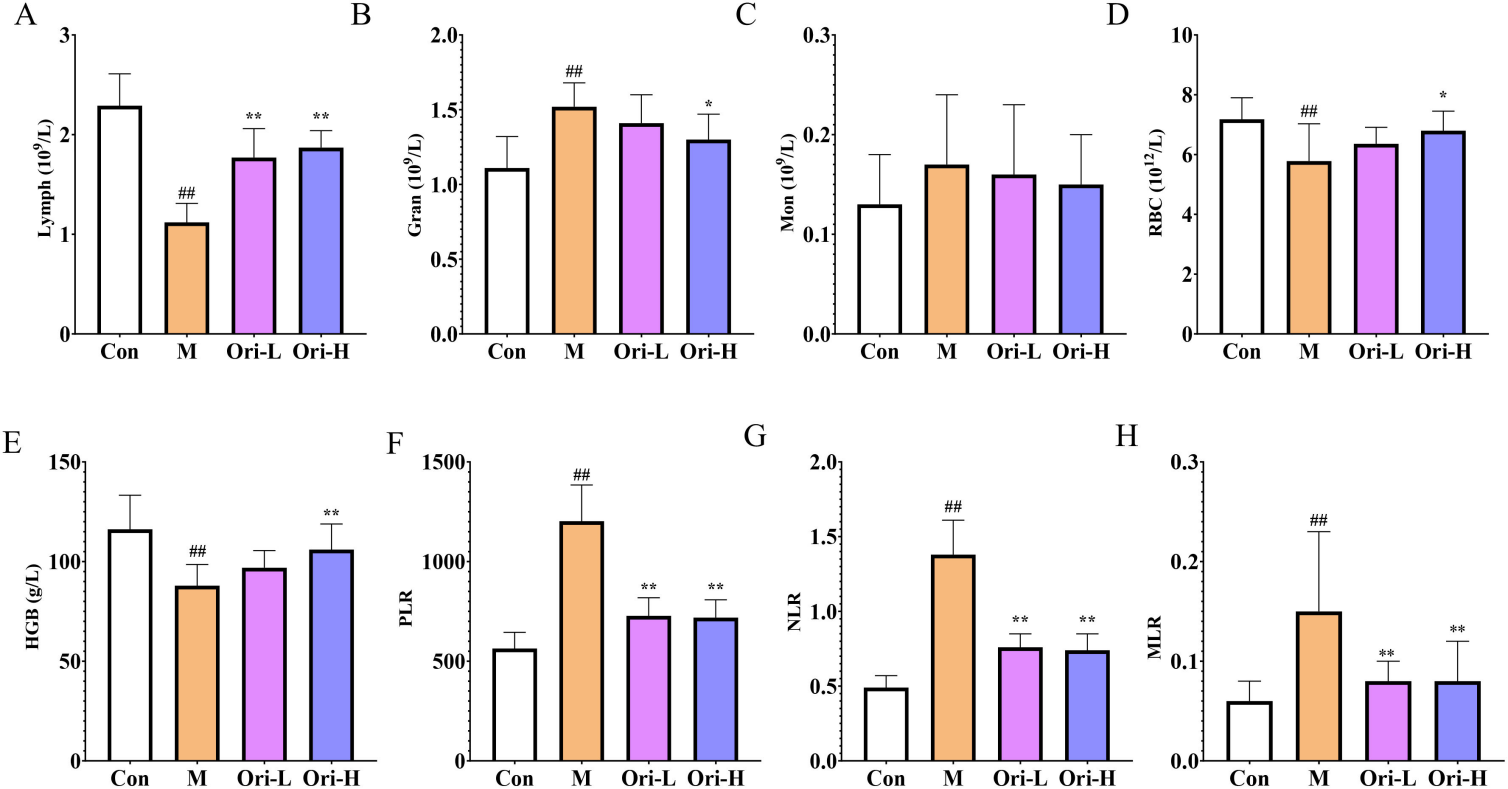
Ori improved peripheral blood cell count. **(A–E)** Lymph, Gran, Mon, RBC, and HGB in peripheral blood. **(F)** platelets to lymph (PLR). **(G)** Neu (Neutrophils) to lymph (NLR). **(H)** Mon to lymph (MLR). Data were expressed means ± SD (*n* = 9-10), the Games-Howell method was used for comparison between two groups (Lymph, Gran, Mon, RBC, HGB, NLR, and MLR), the Bonferroni post-test was used for comparison between two groups (PLR). ^##^ *P* < 0.01 vs the Con; *P < 0.05 vs the M; **P < 0.01 vs the M.

Blood cells are closely related to tumors and can be used for the diagnosis, treatment, and prognosis evaluation of tumor patients. So, we used the blood cell analytical apparatus to detect the count of various types of blood cells. It can be seen from **Figure 3A**, relative to the Con group, Lymph was significantly lower in the M group (*P <* 0.01). Relative to the M group, Lymph showed a significant increase in the Ori-L and Ori-H groups (*P* < 0.01). As shown in **Figure 3B**, relative to the Con group, Gran was significantly increased in the M group (*P <* 0.01). Relative to the M group, Gran showed a significant decrease in the Ori-H group (*P* < 0.05), and Gran showed a decreasing trend in the Ori-L group (*P* > 0.05). As we can see from **Figure 3C**, there was no obvious change in Mon between the groups.

In addition, anemia is one of the complications of most malignant tumors. Tumor, especially EC, has a serious impact on patients’ diet and water intake, resulting in insufficient nutrient intake, leading to a decrease in the body’s RBC and HGB levels, making anemia more likely to occur (25). It can be seen from **Figures 3D, E**, relative to the Con group, RBC and HGB were significantly lower in the M group (*P <* 0.01). Relative to the M group, the peripheral blood RBC and HGB in the Ori-L group showed an increasing trend (*P* > 0.05). And the peripheral blood RBC and HGB in the Ori-H group were increased (*P* < 0.05, *P* < 0.01, respectively).

NLR, MLR, and PLR are often used to evaluate the level of systemic inflammation in tumor patients. Based on this, relevant indicators were statistically analyzed, and the results are shown in **Figures 3F–H**. Relative to the Con group, MLR, NLR, and PLR were significantly increased in the M group (*P <* 0.01), indicating that the mice with EC induced by 4-NQO showed obvious inflammatory reactions. Compared with the M group, MLR, NLR, and PLR were significantly decreased in the Ori-L and Ori-H groups (*P <* 0.01), indicating that Ori could inhibit the inflammatory reaction in mice with EC.

### Ori inhibited the activation of TLR4/NF-κB/NLRP3 inflammasome pathway of EC mice induced by 4-NQO

**Figure 4:**
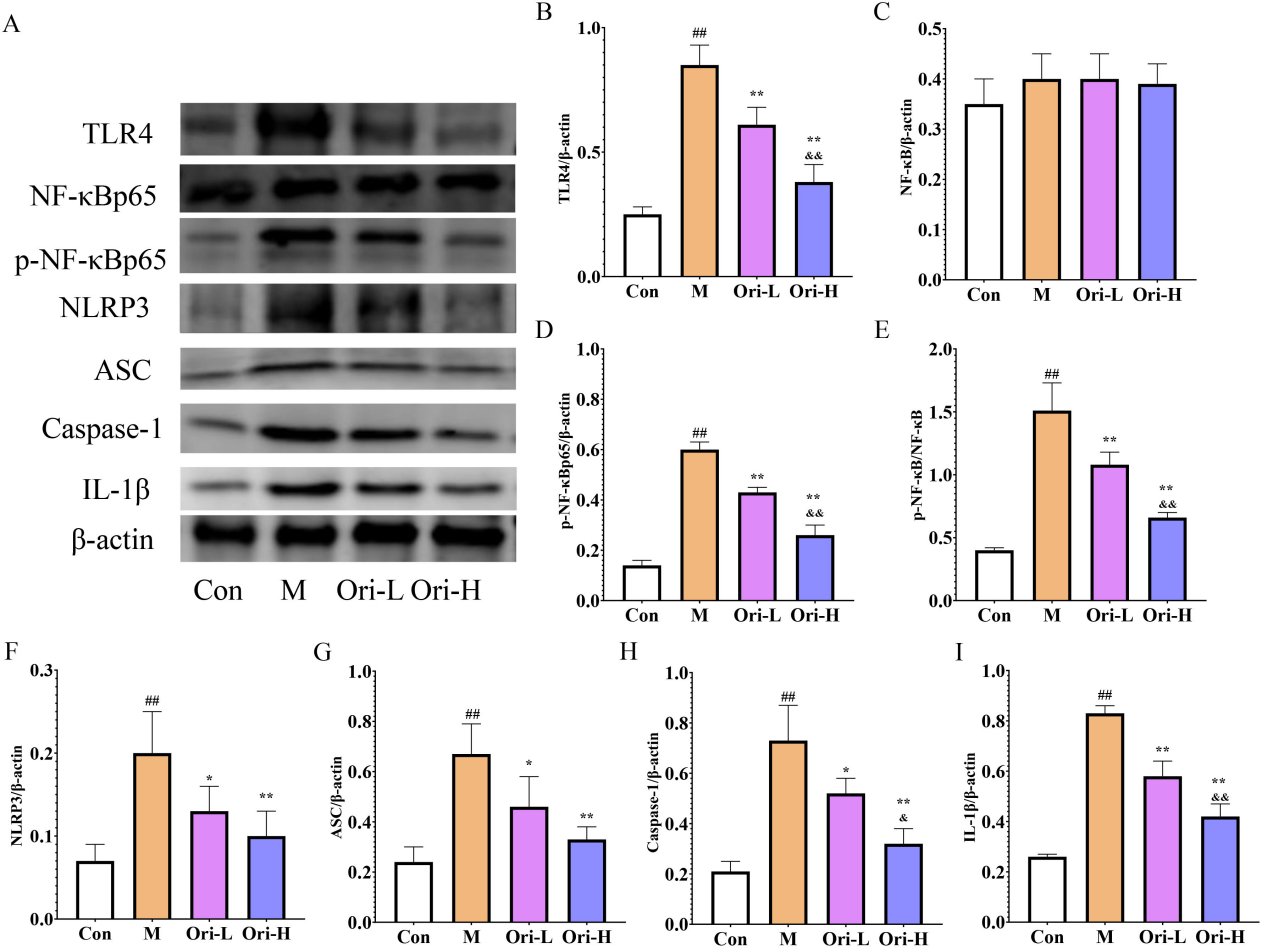
Effects of Ori on TLR4/NF-κB/NLRP3 signaling pathways in the esophageal tissue of 4-NQO mediated EC mice. **(A)** Representative WB and densitometric quantification for **(B)** TLR4, **(C)** NF-κB, **(D)** p-NF-κB, **(E)** p-NF-κB/NF-κB, **(F)** NLRP3, **(G)** ASC, **(H)** Caspase-1, **(I)** IL-1β. Data were expressed means ± SD. n=3 biological repeats. The Games-Howell method was used for comparison between two groups. ^##^ *P* < 0.01 vs the Con; *P < 0.05 vs the M; **P < 0.01 vs the M; ^&^ *P* < 0.05 Ori-H vs the Ori-L, ^&&^ *P* < 0.01 Ori-H vs the Ori-L.

Based on the results of blood cell count, 4-NQO induced severe inflammatory reaction in mice with EC. Combining the TLR4/NF-κB/NLRP3 inflammasome pathway, which is a key link in the inflammatory response, we used WB to detected relevant indicators in esophageal tissue. It can be seen from **Figure 4A** that the expression of TLR4, NLRP3, NF-κB, Caspase-1, p-NF-κB, ASC, and IL-1β proteins varies in the esophageal tissues of different groups of mice. As shown in **Figures 4B–E**, relative to the Con group, the TLR4, p-NF-κB, and p-NF-κB/NF-κB proteins in the esophageal tissue of the M group were increased significantly (*P* < 0.01). Relative to the M group, protein expression of TLR4, p-NF-κB, and p-NF-κB/NF-κB in the esophageal tissue of the Ori-L and Ori-H groups was significantly decreased (*P* < 0.01). Relative to the Ori-L group, protein expression of TLR4, p-NF-κB, and p-NF-κB/NF-κB in the esophageal tissue of the Ori-H group was significantly decreased (*P* < 0.01). There was no statistically significant difference in NF-κB protein among the groups.

As shown in **Figures 4F–I**, relative to the Con group, the NLRP3, Caspase-1, ASC, and IL-1β proteins in the esophageal tissue of the M group were significantly increased (*P* < 0.01). Relative to the M group, the protein expression of NLRP3, ASC, and Caspase-1 was significantly decreased (*P <* 0.01), and the expression of NLRP3 was decreased in the Ori-L group (*P <* 0.05). The NLRP3, Caspase-1, ASC, and IL-1β in the Ori-H group were very significantly decreased (*P* < 0.01). Relative to the Ori-L group, the protein expression of Caspase-1 and IL-1β in the esophageal tissue of the Ori-H group was significantly decreased (*P* < 0.05, *P* < 0.01). The experimental results indicate that Ori may reduce the body’s inflammatory response by inhibiting the activation of TLR4/NF-κB/NLRP3 inflammasome in the esophageal tissues of mice with EC.

### Ori reduced the secretion of inflammatory factors in 4-NQO induced EC

**Figure 5:**
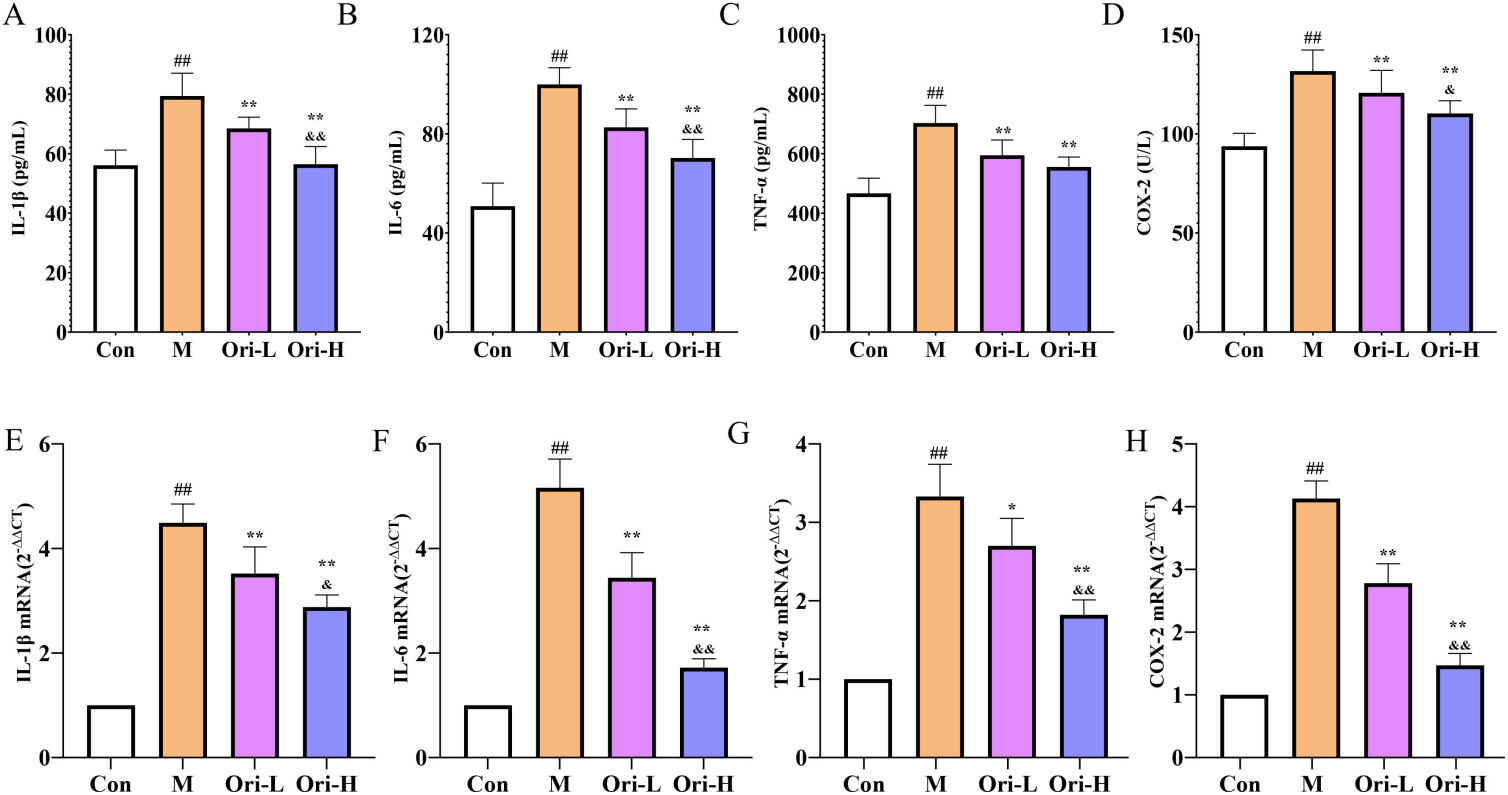
Effects of Ori on the levels of inflammatory factors IL-1β, COX-2, TNF-α, and IL-6 in serum and esophageal tissues of 4-NQO mediated EC mice. **(A–D)** Levels of IL-1β, TNF-α, IL-6, and COX-2 in serum. Data were expressed means ± SD (*n* = 9-10). **(E–H)** The IL-1β, TNF-α, IL-6, and COX-2 mRNA levels in esophageal tissues. Data were expressed means ± SD. n= 3 biological repeats. The Games-Howell method was used for comparison between two groups. ^##^ *P* < 0.01 vs the Con; *P < 0.05 vs the M; **P < 0.01 vs the M; ^&^ *P* < 0.05 Ori-H vs the Ori-L, ^&&^ *P* < 0.01 Ori-H vs the Ori-L.

Subsequently, ELISA and RT-PCR were used to detect the levels of IL-1β, COX-2, TNF-α, and IL-6 in serum and esophageal tissue, respectively. As shown in **Figures 5A–D**, relative to the Con group, the serum levels of IL-1β, COX-2, TNF-α, and IL-6 were significantly increased in the M group (*P <* 0.01). Relative to the M group, the serum levels of COX-2, TNF-α, and IL-6 were significantly decreased in the Ori-L and Ori-H groups (*P <* 0.01). Relative to the Ori-L group, the serum levels of IL-1β, COX-2, and IL-6 were significantly decreased in the Ori-H group (*P* < 0.05, *P* < 0.01).

As shown in **Figures 5E-H**. Relative to the Con group, the mRNA levels of IL-1β, COX-2, TNF-α, and IL-6 were significantly increased in the esophagus tissue of the M group (*P <* 0.01). Relative to the M group, the mRNA levels of IL-1β, COX-2, and IL-6 were significantly decreased (*P <* 0.01), and the mRNA level of TNF-α was decreased in the esophagus tissue of the Ori-L group (*P <* 0.05). The mRNA levels of IL-1β, COX-2, TNF-α, and IL-6 were decreased significantly in the esophagus tissue of the Ori-H group (*P <* 0.01). Relative to the Ori-L group, the mRNA levels of IL-1β, COX-2, TNF-α, and IL-6 were significantly decreased in the Ori-H group (*P* < 0.05, *P* < 0.01). The above results suggest that Ori can not only inhibit systemic inflammation levels in mice with EC, but also suppress local inflammatory responses in esophageal tissue.

### Ori affected the expression of PCNA, Ki67, Bcl-2, and Bax mRNA in the esophageal tissue of EC mice induced by 4-NQO

**Figure 6:**
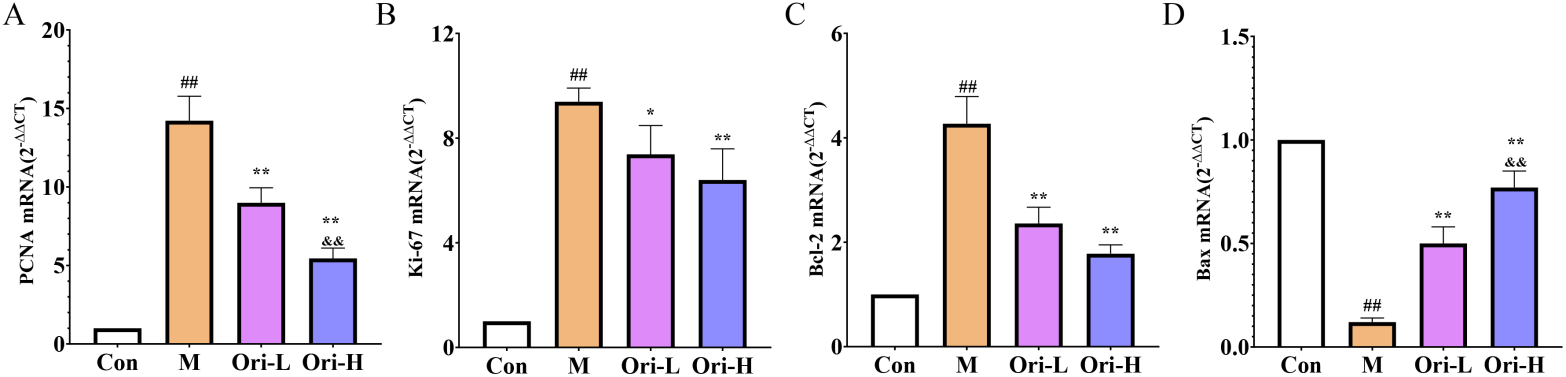
Effects of Ori on the expression of PCNA, Ki67, Bcl-2, and Bax mRNA in the esophageal tissue of 4-NQO induced EC mice. **(A–D)** The PCNA, Ki67, Bcl-2, and Bax mRNA levels in esophageal tissues. Data were expressed means ± SD. n=3 biological repeats. The Games-Howell method was used for comparison between two groups. ^##^ *P* < 0.01 vs the Con; *P < 0.05 vs the M; **P < 0.01 vs the M; ^&&^ *P* < 0.01 Ori-H vs the Ori-L.

As shown in **Figures 6A–D**, relative to the Con group, the expression of PCNA, Ki67, and Bcl-2 mRNA was significantly increased, and Bax mRNA was significantly decreased in the esophagus tissue of the M group (*P <* 0.01). Relative to the M group, the expression of PCNA, Ki67, and Bcl-2 mRNA was significantly decreased, and Bax mRNA in the esophagus tissue of the Ori-L and Ori-H groups was significantly increased (*P <* 0.01). Relative to the Ori-L group, the mRNA level of PCNA was significantly decreased, and Bax was significantly increased in the Ori-H group (*P* < 0.01).

### Ori affected the protein expression of E-cadherin, N-cadherin, GSK3β, and p-GSK3β in the esophageal tissue of EC mice induced by 4-NQO

**Figure 7:**
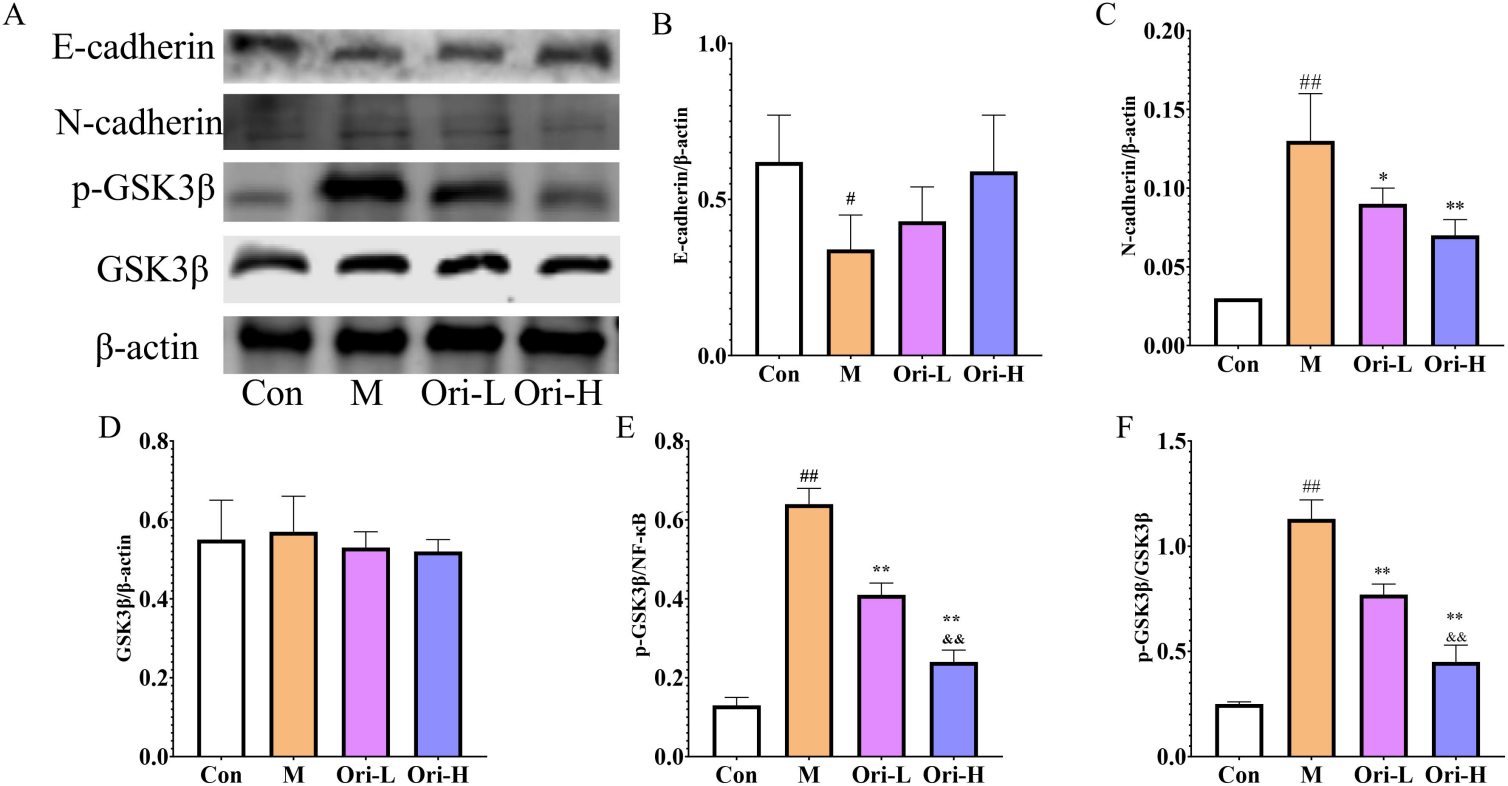
Effects of Ori on the expression of E-cadherin, N-cadherin, GSK3β, and p-GSK3β proteins in the esophageal tissue of 4-NQO induced EC mice. **(A)** Representative WB and densitometric quantification for **(B)** E-cadherin, **(C)** N-cadherin, **(D)** GSK3β, **(E)** p-GSK3β, **(F)** p-GSK3β/GSK3β. Data were expressed means ± SD. n=3 biological repeats. The Games-Howell method was used for comparison between two groups. ^##^ *P* < 0.01 vs the Con; *P < 0.05 vs the M; **P < 0.01 vs the M; ^&&^ *P* < 0.01 Ori-H vs the Ori-L.

From **Figure 7A**, it can be observed that, except for GSK3β, the expression levels of E-cadherin, N-cadherin, and p-GSK3β proteins differ among the esophageal tissues of various groups of mice. As shown in **Figures 7B–F**, relative to the Con group, the expression levels of E-cadherin, N-cadherin, p-GSK3β, and p-GSK3β/GSK3β proteins in the esophageal tissue of the M group mice was significantly increased (*P* < 0.05 or *P* < 0.01). Relative to the M group, the expression levels of N-cadherin, p-GSK3β, and p-GSK3β/GSK3β were significantly decreased in the esophageal tissues of both Ori-L and Ori-H groups (*P* < 0.05 or *P* < 0.01), while the expression level of E-cadherin showed an upward tendency, though not reaching statistical significance (*P* > 0.05). Relative to the Ori-L group, the expression levels of p-GSK3β and p-GSK3β/GSK3β were significantly decreased in the esophageal tissues of the Ori-H group (*P* < 0.05, *P* < 0.01).

## Discussion

EC is not only a common gastrointestinal malignancy, but also has a poor prognosis and high mortality rate. Although a combination therapy strategy has been developed, the quality of life and prognosis of esophageal cancer patients are still not optimistic. Early surgical resection has the best effect, but the early symptoms of EC are not typical, and most cases are diagnosed in the middle to late stage, missing the optimal surgical treatment period. Moreover, postoperative infection can lead to wound deterioration and increase the risk of developing complications. Although therapies such as targeted therapy, chemotherapy, immunotherapy and radiotherapy can, to some extent, limit tumor growth, most of them are difficult to distinguish between normal and malignant cells, leading to non-specific cytotoxicity and side effects (26). Therefore, the demand for efficient and low toxicity drugs is still urgent. TCM plays an important role in the treatment of EC. It often takes into account clinical classification, staging, and symptoms, and provides individualized treatment for patients from the perspectives of clearing heat and removing toxicity, dispelling hardness and resolving stagnation, promoting blood circulation and removing blood stasis, strengthening the body and nourishing the vital essence, reducing reflux, and regulating the stomach function. To a certain extent, it can help tumor patients “carry tumors and prolong life”, and play a significant role in improving symptoms, enhancing immunity, improving quality of life, and prolonging survival. Ori is an active tetracyclic diterpenoid compound in *Rabdosia rubescens (Hemsl.)*, which is the main anti-cancer and anti-inflammatory component of *Rabdosia rubescens (Hemsl.)*. More and more studies have shown that Ori can exert anti-tumor effects by regulating various pathways such as PI3K, Bcl-2/Bax, MAPK, p53/p21, and microRNAs (27). In addition to anti-tumor mechanisms such as cell cycle arrest, induction of tumor cell apoptosis, reversal of chemoresistance, and inhibition of cancer cell invasion, Ori also has strong anti-inflammatory effects (28, 29). The anti-tumor mechanisms of Ori, including inducing apoptosis, inhibiting invasion, and metastasis, are directly or indirectly related to its anti-inflammatory effects (30, 31). Recent studies have shown that the activation of NLRP3 inflammasomes is involved in the occurrence and development of EC (9, 10). He et al. (21) showed that Ori can inhibit the activation of inflammasomes by binding to the 279 cysteine covalent bond in the NLRP3 NACHT domain through carbon double bonds. However, it is not yet known whether Ori can exert its anti-EC effect by inhibiting the activation of NLRP3 inflammasomes.

In the course of EC, due to the consumption of energy by tumor cells and the patients’ long-term insufficient nutrient intake, the quality of life can be seriously reduced. Dysphagia is one of the common symptoms in patients with EC. Patients experience obstruction when swallowing water or food, and in severe cases, they are unable to eat directly, which affects the body’s intake of nutrients, reduces the patient’s quality of life, and can easily lead to a shortened survival period (32). Patients with advanced EC mainly rely on nasal feeding, and a few can consume liquid food on their own. According to reports, dysphagia is the most severe symptom in patients 1-3 days after surgery; Within 2 years after surgery, the incidence of dysphagia in patients is as high as 58%, increasing the risk of aspiration pneumonia and death (33, 34). As one of the indicators reflecting the nutritional status and disease status of the human body, weight has been preliminarily confirmed by clinical studies to be associated with esophageal cancer. Specifically, individuals who have lost ≥ 2 kg in weight within 6 years have a significantly increased risk of esophageal cancer mortality, and this association is not affected by age, gender, or baseline BMI status (35). Therefore, reducing the degree of cancer in esophageal tissue and improving swallowing difficulties can ensure food safety, improve eating experience, reduce weight loss, and improve quality of life. In the mouse model of EC, we evaluated the degree of cancer transformation and swallowing function by observing diet and water intake and conducting HE staining, and further observed body weight indicators. The results showed that Ori can reduce the degree of esophageal tissue carcinogenesis in mice with EC, increase diet, water intake, and body weight, indicating that it can alleviate swallowing difficulties to a certain extent and improve quality of life. According to reports, Gran and Mon are positively correlated with the malignancy of tumors and can enhance the invasion and migration ability of tumor cells; Lymph can recognize and kill tumor cells, thereby inhibiting tumor proliferation, and is the main executor of immune function in the anti-tumor process. Tumor-related Gran is the core of acute inflammation and participates in tumor-related chronic inflammation. Mon can be recruited to the tumor site and “domesticated” by cancer cells into tumor-related macrophages, which are the main components of the tumor microenvironment. Tumor-related macrophages have immunosuppressive functions and can help tumor cells spread and metastasize, thereby promoting tumor malignant progression (36, 37). NLR, MLR, and PLR are often used to evaluate the level of systemic inflammation in cancer patients, and are related to the degree of malignancy of EC, neoadjuvant chemotherapy, immunotherapy, chemotherapy effect, the incidence of adverse events, and prognosis evaluation (38, 39). We found that Ori can increase Lymph in the peripheral blood of EC mice, reduce Gran, NLR, MLR, and PLR, suggesting that it can improve the disorder of blood cells, enhance anti-tumor function, and inhibit systemic inflammatory response. Moreover, NLR, MLR, and PLR are clinically readily available indicators and cause little trauma to patients. In future clinical trials, the clinical efficacy of oridonin can be evaluated based on changes in blood cell ratios (NLR, PLR, and MLR) before and after intervention.

Chronic inflammation is an important event in the occurrence and development of tumors, and cancer-related inflammation has been identified as the seventh characteristic of tumors. NLRP3 inflammasomes play a central role in the inflammatory response and have been confirmed to be involved in EC (9, 10). The NLRP3 protein is a receptor of inflammasomes, which, together with the regulator ASC and effector Caspase-1, regulate the maturation and release of inflammatory factor IL-1β, and further induces the release of downstream inflammatory factors TNF-α and IL-6. Under physiological conditions, locally low levels of cytokines such as IL-1β, TNF-α, and IL-6 can participate in the normal immune response of the body, while abnormal expression can lead to systemic inflammatory reactions in the body (40). Among them, IL-1β can promote the migration and infiltration of esophageal squamous cell carcinoma cells, and increase the malignant cytological behavior of EC cells (41). IL-6 is highly expressed in the serum of EC patients, and the expression level of IL-6 in the serum significantly increases with the increase of tumor stage. Patients with lymph node metastasis, deeper tumor infiltration depth, and higher TNM stage have higher expression levels of IL-6 in the serum (42). TNF-α is a pro-inflammatory cytokine that can promote the migration of EC cells by inducing MMP9 expression (43). The results of WB, ELISA, and RT-PCR showed that Ori can reduce the expression of NLRP3, ASC, Caspase-1, and IL-1β proteins in esophageal tissue, reduce the levels of serum inflammatory factors IL-1β, TNF-α, and IL-6, as well as the levels of IL-1β, TNF-α, and IL-6 mRNA in esophageal tissue, indicating that Ori can inhibit the inflammatory response of EC mice by inhibiting the activation of NLRP3 inflammasomes. As reported, the activation of NLRP3 inflammasomes is regulated by the TLR4/NF-κB signaling pathway. The binding of TLR4 with LPS can activate its downstream IκB kinase, causing IκαB to phosphorylate and degrade, thereby activating NF-κB, promoting the expression and assembly of NLRP3, ASC, and pro-Caspase-1 to form NLRP3 inflammasomes. The activated NLRP3 inflammasome promotes cell pyroptosis, causing swelling and pore formation, thereby releasing inflammatory factors such as IL-1β, TNF-α, and IL-6 (44). On the basis of determining that Ori can inhibit the activation of NLRP3 inflammasomes, we continued to detect the expression of upstream related proteins in this pathway. The results showed that TLR4 and p-NF-κB proteins were highly expressed in the esophageal tissue of EC mice, while Ori can reduce the expression levels of TLR4 and p-NF-κB proteins, indicating that Ori inhibits NLRP3 inflammasome activation by inhibiting the TLR4/NF-κB signaling pathway.

**Figure 8:**
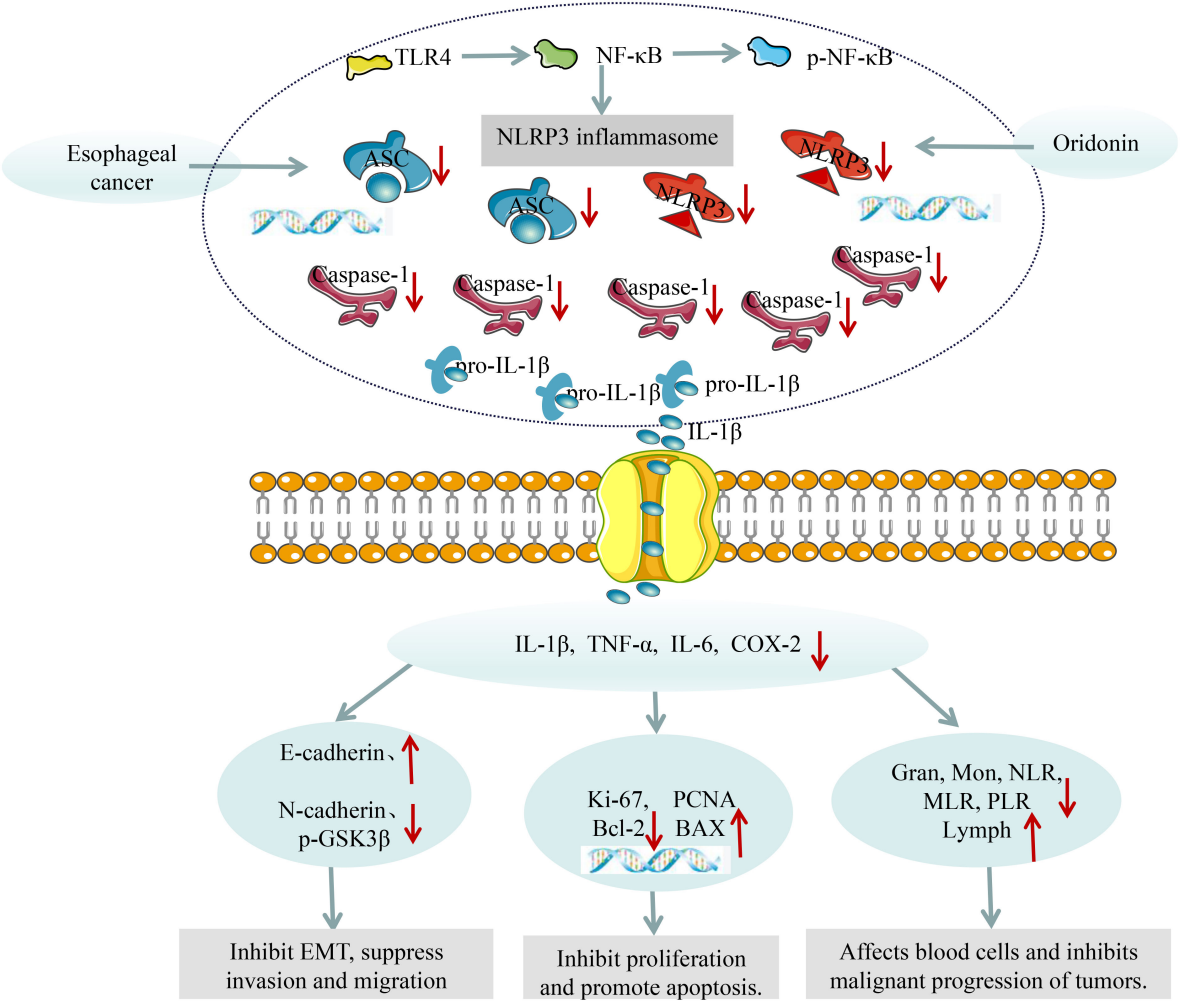
Effects of Ori on EC.

The inflammatory microenvironment formed by inflammatory factors such as IL-1β, TNF-α, and IL-6 can promote the proliferation and interstitial transformation of tumor cells, thereby inducing their invasion and metastasis to adjacent or distant tissues. Ki-67 antigen is a specific antigen related to cell proliferation, which is not expressed in the G0 phase of the cell cycle but begins to be expressed in the G1 phase, continues to be expressed in G1, S, G2, and M phases, and rapidly decreases after mitosis, which is a widely used indicator for evaluating the proliferative activity of tumor cells (45). PCNA is a kind of auxiliary protein of DNA polymerase, which is indispensable in the process of cell DNA replication. The expression level of PCNA is low in the G0 phase, increases in the G1 phase, and reaches the peak in the S phase. It can effectively evaluate the status of tumor cell DNA synthesis and the level of cell proliferation dynamics, and is often used for tumor cell proliferation detection (46). The decrease of E-cadherin and the increase of N-cadherin and p-GSK3β are markers of tumor cell EMT, which is an important prerequisite for tumor cell invasion and metastasis. Our results showed that Ori can reduce the expression of PCNA and Ki-67 mRNA, as well as N-cadherin, p-GSK3β protein in esophageal tissue, while increase E-cadherin protein expression. Bcl-2 and Bax are classical factors in the Bcl-2 family of apoptosis-related proteins, with opposite functions. Bcl-2 can resist cell apoptosis, while Bax can antagonize the protective effect of Bcl-2 on cancer cells (47, 48). After NLRP3 inflammasome activation, Pro-Caspase-1 will be activated and cleaved to form active Caspase-1. Activated Caspase-1 cleaves GSDMD and IL-1β and IL-18 precursors, leading to cell pyroptosis and ultimately inducing and amplifying inflammatory responses (49). After cell pyroptosis occurs, a large amount of inflammatory factors are released, which facilitates the formation of the tumor microenvironment and protects tumor cells from apoptosis (50). Our results indicate that Ori can reduce Bcl-2 mRNA levels and increase Bax mRNA levels in esophageal tissue of mice with esophageal cancer, suggesting that Ori can promote tumor cell apoptosis by inhibiting NLRP3 inflammasome activation. In summary, we speculate that Ori can alleviate inflammation and ultimately improve EC by inhibiting TLR4/NF-κB/NLRP3 inflammasomes (**Figure 8**).

However, there were several limitations to this experiment. For example, this study only conducted pharmacological experiments and did not conduct *in vivo* knockdown or overexpression bidirectional validation of the intervention effect of Ori on TLR4/NF-κB/NLRP3 in esophageal cancer mice, as well as its downstream effects. Secondly, although this study proposed that oridonin may inhibit the invasion, metastasis, and apoptosis of esophageal cancer cells by intervening in TLR4/NF-κB/NLRP3, no correlation analysis was conducted and lacked *in vitro* experimental verification. Finally, the model used in this study is a chemically induced esophageal cancer model. Although 4-NQO induced esophageal cancer is recognized as a model consistent with clinical progression of esophageal cancer, it did not utilize patient-derived xenografts, and its clinical relevance is limited. These will be the focus of our next work. Moreover, we will pay more attention to the close connection between basic research and clinical practice, such as using human tumor cells for *in vitro* and *in vivo* experiments.

## Data Availability

The original contributions presented in the study are included in the article/supplementary material. Further inquiries can be directed to the corresponding authors.
